# Varying foraging patterns in response to competition? A multicolony approach in a generalist seabird

**DOI:** 10.1002/ece3.1884

**Published:** 2016-01-20

**Authors:** Anna‐Marie Corman, Bettina Mendel, Christian C. Voigt, Stefan Garthe

**Affiliations:** ^1^ Research and Technology Centre (FTZ)University of KielHafentörn 125761BüsumGermany; ^2^ Leibniz Institute for Zoo and Wildlife Research (IZW)Alfred‐Kowalke‐Straße 1610315BerlinGermany

**Keywords:** Foraging strategy, GPS tracking, intraspecific competition, *Larus fuscus*, stable isotope analyses, utilization distribution

## Abstract

Reducing resource competition is a crucial requirement for colonial seabirds to ensure adequate self‐ and chick‐provisioning during breeding season. Spatial segregation is a common avoidance strategy among and within species from neighboring breeding colonies. We determined whether the foraging behaviors of incubating lesser black‐backed gulls (*Larus fuscus*) differed between six colonies varying in size and distance to mainland, and whether any differences could be related to the foraging habitats visited. Seventy‐nine incubating individuals from six study colonies along the German North Sea coast were equipped with GPS data loggers in multiple years. Dietary information was gained by sampling food pellets, and blood samples were taken for stable isotope analyses. Foraging patterns clearly differed among and within colonies. Foraging range increased with increasing colony size and decreased with increasing colony distance from the mainland, although the latter might be due to the inclusion of the only offshore colony. Gulls from larger colonies with consequently greater density‐dependent competition were more likely to forage at land instead of at sea. The diets of the gulls from the colonies furthest from each other differed, while the diets from the other colonies overlapped with each other. The spatial segregation and dietary similarities suggest that lesser black‐backed gulls foraged at different sites and utilized two main habitat types, although these were similar across foraging areas for all colonies except the single offshore island. The avoidance of intraspecific competition results in colony‐specific foraging patterns, potentially causing more intensive utilization of terrestrial foraging sites, which may offer more predictable and easily available foraging compared with the marine environment.

## Introduction

**Figure 1:**
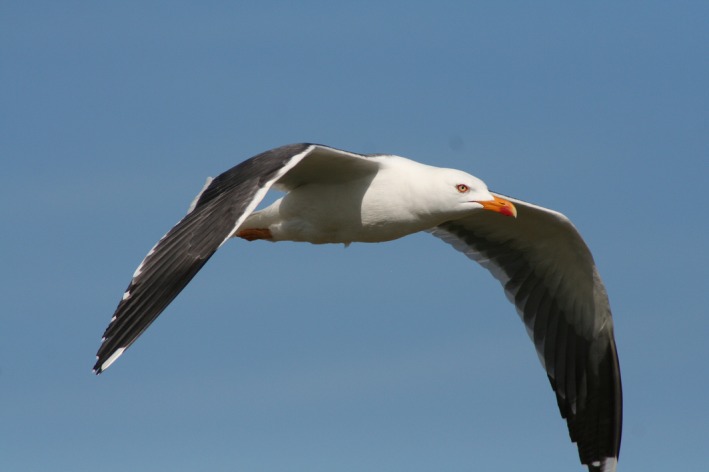
Adult lesser black‐backed gull (*Larus fuscus*).

Colonial seabirds are central‐place foragers during the breeding period and therefore depend on continuous and sufficient availability of prey within accessible distances of their breeding colony (Wittenberger and Hunt 1985). Optimal foraging theory suggests that animals should use the minimum traveling distances necessary to satisfy their energy demands (Schoener 1971). Visiting more distant sites will thus only be profitable if the prey is of higher quality, more abundant, or more easily available than at sites closer to the colony (Houston and McNamara 1985; Harding et al. 2013). Density‐dependent competition, both within and between adjacent colonies with overlapping foraging ranges, represents another limiting factor for colonial breeders (e.g., Cairns 1989; Gaston et al. 2007). In accordance with Ashmole's theory, larger colonies have larger foraging ranges (Lewis et al. 2001) because of food depletion within the immediate vicinity of the colony (Ashmole 1963). Recent studies found that foraging sites of individuals from neighboring colonies were spatially segregated to minimize intraspecific competition for the same resources (e.g., Grémillet et al. 2004; Wakefield et al. 2013). Seabird conspecifics from different breeding colonies may thus either occupy different foraging spaces and/or foraging niches, especially given that prey distribution may also vary around different colonies. Foraging niche width may be derived from the variation of isotopic values (“isotopic‐niche width” proposed by Newsome et al. (2007); that is, area spanned by isotopic values as coordinates) calculated from stable isotope analyses (SIA) of carbon and nitrogen. SIA is a common tool for integrating trophic information from different tissues over different time periods (Bearhop et al. 2004; Layman et al. 2012) and can be used to quantify foraging strategies at both the individual and population levels (Newsome et al. 2007). Several studies have dealt with the short‐ and long‐term consistencies of the variation of isotopic values at the individual level (e.g., Woo et al. 2008; Ceia et al. 2014). However, existing isotopic studies at the colony level have often been based on visual observations of seabirds' foraging behavior. The possibility of tracking individuals from the corresponding colonies has often been dismissed because of technical constraints (e.g., Forero et al. 2002), or studies have focused on interspecific seabird communities (e.g., Harding et al. 2013; Bodey et al. 2014b). GPS data loggers currently provide a common tool for analyzing the year‐round foraging behavior of seabirds (e.g., Shamoun‐Baranes et al. 2012). However, the sample size of tracking studies (of the same species at different breeding sites) is often small, and many studies have therefore focused on individual foraging patterns, instead of considering a multicolony approach. Wakefield et al. (2013) reported that the colony‐specific home ranges of tracked northern gannets (*Morus bassanus*) were strongly related to density‐dependent, intraspecific competition. Gannets feed largely on pelagic shoaling fish and thus exclusively use the marine habitat for foraging. In contrast, we investigated the foraging behaviors of the omnivorous lesser black‐backed gull (LBBG; *Larus fuscus*; Fig. 1), which is a common opportunistic seabird species that utilizes a broad range of foraging habitats both at sea and on land (e.g., Kubetzki and Garthe 2003). We conducted a multiyear tracking study at six different breeding colonies in the southern North Sea and examined colony‐specific foraging areas by combining distributional patterns with conventional and isotopic dietary data from tracked individuals. All breeding colonies were located on islands with direct access to the open sea. Given that colony size and location are known to affect the foraging behavior of seabirds, we expected differences in foraging behaviors in relation to the size of the colony and its location, in terms of distance from the mainland. According to foraging theory, we hypothesized that there would be clearly segregated foraging patterns with little spatial overlap among colonies, despite the close proximity of some colonies to each other. We anticipated similar, but less distinct, results for dietary segregation, given that individual diet preferences may vary. We also determined whether foraging habitat type (sea or land) affected the foraging behaviors of LBBGs from different colonies. We predicted that the avoidance of density‐dependent competition might favor the use of marine and terrestrial habitats to different extents.

## Materials and Methods

### Fieldwork

**Figure 2:**
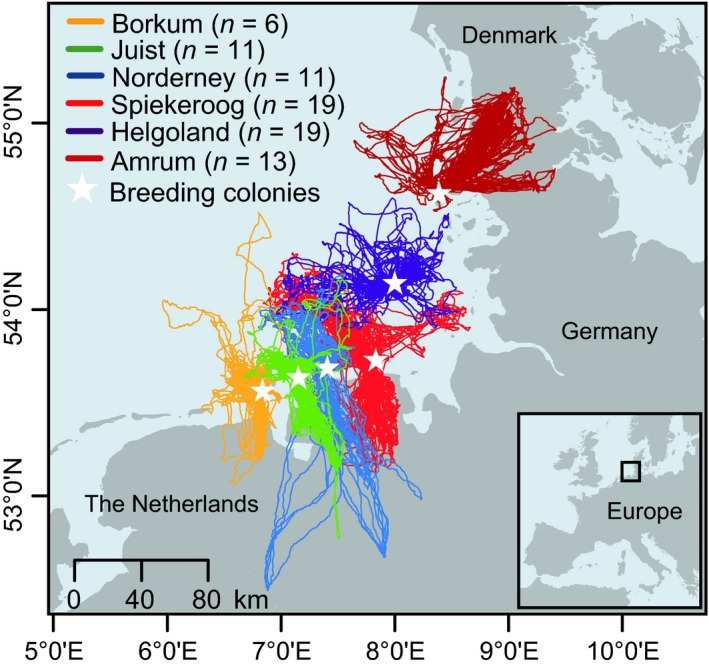
Foraging trips of all tracked *Larus fuscus* (*n* = 79) in the southern North Sea. White stars represent the location of the breeding colonies. Different colors indicate birds from different breeding colonies.

Incubating LBBGs were caught at six German breeding colonies from 2008 to 2013 (Table 1, Fig. 2): the East and North Frisian Wadden Sea islands Borkum (BO), Juist (JU), Norderney (NO), Spiekeroog (SP), and Amrum (AM), and the offshore island of Helgoland (HE) (see Table S1 for distances between breeding colonies). All gulls were caught using walk‐in traps placed above their nests. Seventy‐nine of 103 captured and equipped LBBGs with appropriate data sets were successfully recaptured after approximately 16 days to remove the GPS devices. The 24 remaining individuals could not be recaptured (*n* = 18), had lost (*n* = 1) or malfunctioning (*n* = 2) devices, or incomplete data sets with only one incomplete trip (*n* = 3). Thus, 79 data sets comprising 838 foraging trips were available for the analyses of foraging patterns.

**Table 1:** Study sites, study periods, and sample size of tagged *Larus fuscus*

Colony	Coordinates	Area (km²)	No. of breeding pairs (status)	Study period	Distance to mainland (km)	No. of birds (evaluable data sets)	No. of foraging trips per individual (mean ± SD)
AM	54°41′N, 8°20′E	20.46	ca. 10,000^a^ (2012)	13–31 May 2011	24	6	5.5 ± 5.4
				19 May–4 June 2012		7	12.4 ± 1.8
BO	53°43′N, 7°18′E	30.74	ca. 1500^b^ (2012)	20 May–4 June 2012	17	6	13.8 ± 2.3
HE	54°11′N, 7°53′E	1.70	ca. 600^c^ (2013)	24 May–3 June 2008	45	4	5.8 ± 2.6
				2 May–3 June 2009		5	6.0 ± 4.1
				18–30 May 2010		5	5.4 ± 1.5
				17–28 May 2011		5	12.0 ± 4.3
JU	53°40′N, 7°04′E	16.43	ca. 1000^b^ (2012)	16 May–4 Jun 2013	10	11	17.4 ± 10.2
NO	53°43′N, 7°18′E	26.29	ca. 4000^b^ (2012)	16 May–3 June 2013	4	11	8.7 ± 8.6
SP	53°46′N, 7°42′E	18.25	ca. 8000^b^ (2012)	14–23 May 2009	7	6	5.7 ± 2.0
				16 May–3 June 2010		8	13.3 ± 3.0
				19 May–5 June 2012		7	12.0 ± 4.3

a Verein Jordsand e.V. (unpubl. data).

b Wadden Sea National Park Administration of Lower Saxony (unpubl. data).

c Island Station of the Institute of Avian Research “Vogelwarte Helgoland” (unpubl. data). Numbers of breeding pairs are overall numbers for the corresponding islands.

### GPS telemetry

Streamlined GPS data loggers (Earth & Ocean Technologies, Kiel, Germany; Catnip Technologies, Hong Kong, China) were attached to the base of the four innermost tail feathers using TESA tape (Beiersdorf AG GmbH, Hamburg, Germany). The total weights of the two types of attached devices were 26 and 30 g, respectively, which were 3.3% (range: 2.6–4.3%) and 3.6% (range: 3.0–4.3%) of the average body mass of most equipped LBBGs (mean ± SD: 797.4 ± 100.1 g; range: 609–976 g; *n*_birds_ = 58). The recording intervals of the devices were set to 2 min, except in individuals caught at SP in 2010, which were tracked every 3 min. Eight devices applied at JU and NO had a special schedule with a main recording interval of 5 min.

### Instrumentation effect

Total weights of attached devices slightly exceeded the commonly used “3% limit” (e.g., Kenward 2001; Phillips et al. 2003; Barron et al. 2010; but see Vandenabeele et al. 2012; Ludynia et al. 2012), and thus, we conducted additional observations to exclude any effects of the devices on the gulls (details in Data S1).

### Foraging behavior and utilization distribution

Any complete trip made by a gull that was clearly heading out of the relevant breeding colony, where the first and last positions were at the colony, was defined as foraging trip. This study focused on the properties of the foraging trips and we therefore ignored all data during nest attendance and resting within or near the colony. The area close to the breeding colony is frequently used for preening or bathing, and trips for these purposes were assumed to be mostly within a distance of <2 km from the breeding colony and were characterized by few movements and low instantaneous speeds. These trips were excluded from further analyses. The foraging trip characteristics recorded included trip duration and total distance flown and were calculated using the trip package 1.1.18 of R 3.1.1 (R Development Core Team 2014). Each GPS position was assigned visually to either land (i.e., mainland or islands) or sea (ArcGIS 10.0; ESRI 2011) and defined as the proportion of time flying over land for each trip, indicating a visit to either marine (“0”) or terrestrial (“1”) habitats.

Utilization distributions (UD) of the tagged animals were identified for each trip using the biased random bridge approach (BRB; Benhamou 2011; R package adehabitatHR 0.4.11; “BRB” function: grid = 500, radius = 300 m, *h*_min_ = 100 m), taking into account the time dependence between relocations. BRB assumes that animals move following biased random walks and supposes a drift between successive relocations. Individual intensity distribution (ID) and recursion distribution (RD) were also calculated accounting for areas in which the individuals stayed for a long time (ID) and frequently visited areas (RD). Both measurements characterize profitable foraging areas that were exploited intensively, either through increasing residence time (ID) or more visits throughout the study period (RD; Benhamou and Riotte‐Lambert 2012). RD areas contain commuting flights, whereas ID areas did not, and thus represent actual foraging patches. Calculation of IDs and RDs failed for one AM individual resulting in 78 data sets comprising 824 foraging trips for these analyses. We calculated the areas (km²) of 50% and 95% UD, and 30% ID and RD and the corresponding utilization distribution overlap indices (UDOI, Fieberg and Kochanny 2005) between and within colonies using the same R package. The UDOI determines the degree to which two birds share the same space. It ranges between 0 (no overlap) and 1 (complete overlap) as long as UDs are uniformly distributed, but can exceed 1, if the degree of overlap is very high and both UDs are nonuniformly distributed (Fieberg and Kochanny 2005).

### Identification of active foraging

We identified locations of active foraging out of all recorded individual trip locations by overlaying them with the calculated ID areas. Locations at the relevant colony islands as well as locations within 2 km from the breeding colony were excluded from this consideration. All trip locations overlapping with the ID areas were selected as “locations of active foraging” (resulting in 811 foraging trips of the 78 individuals for the corresponding analyses). We summarized the characteristics of these selected active foraging locations per trip and calculated their maximum beeline distance to the breeding colony, the proportion of locations at land/sea (referred to as proportion of foraging at land), and the proportion of locations during the day/night (referred to as proportion of foraging during the day), according to the trip properties noted above.

### Dietary analyses

The prey compositions were compared among the breeding colonies by combined analyses of pellets from the entire colony and SIA of the tagged individuals. Pellets were collected randomly at each study site during the relevant study period and analyzed to the lowest possible taxon according to Kubetzki and Garthe (2003). Only fresh pellets were collected, representing the gulls' diet over the preceding 2–3 days. We compared dietary diversities among colonies using a modification of the Shannon Index (Shannon and Weaver 1949)

$$H^{\prime }=-\sum_{i}p_{i}*\ln\left(p_{i}\right)$$

where H′ is the diversity and *p*_*i*_ is the relative frequency of each dietary component in all pellets per colony.

Carbon and nitrogen SIA is widely used for analyzing the trophic ecology of seabirds (e.g., Inger and Bearhop 2008; Ceia et al. 2014). *δ* ^13^C represents the foraging habitat, with decreasing ratios indicating more terrestrial feeding, while *δ* ^15^N reflects the consumer's trophic level (Inger and Bearhop 2008). Both parameters represent the gulls' diet over the last 3 days (plasma) to 3 weeks (red blood cells, RBCs), depending on the tissue analyzed (Hobson and Clark 1993). We used RBCs in the current analysis because this represented the diet during the active logger periods. Blood samples (max. 0.5 mL corresponding to 0.75% of the total blood volume of the captured gulls; gauge needle: 0.40 × 20 mm; centrifuged within max. 2 h) were taken during recapture from the cutaneous ulnar or brachial vein of 49 tracked individuals (no blood samples available for birds from HE, SP (2009), and one JU individual). Most important prey items identified from pellet analyses of LBBGs (Table S9; this study and, e.g., Kubetzki and Garthe 2003; Schwemmer & Garthe 2005) were caught during research vessel surveys (details in Data S2). RBC samples and prey items were freeze‐dried and homogenized. SIA of RBCs and prey items was conducted at the Leibniz Institute for Zoo and Wildlife Research, Berlin, Germany (details in supplement information S3).

We used trophic differentiation factors (TDFs) (+2.75 for *δ* ^15^N and −0.06 for *δ* ^13^C) (Steenweg et al. 2011) calculated for herring gulls (*L. argentatus*) and great black‐backed gulls (*L. marinus*), because species‐specific TDFs for LBBGs were not available. However, both *Larus* species have similar foraging and feeding ecologies to LBBGs (e.g., Cramp 1983), suggesting that their TDF values were comparable.

### Statistical analyses

All statistical analyses were carried out using R 3.1.1. Linear mixed models (LMM; Faraway 2006) based on the REML estimation were performed (R package lme4 1.1‐6). Trip duration, total distance flown, areas of 95% and 50% UDs, 30% IDs and RDs, including the relevant intracolonial overlaps were used as response variables in these separate models. Analyses of active foraging considered three response variables: maximum distance to the relevant breeding colony and the proportions of active foraging at land/sea and during the day/night (day: 05:00–21:59 CEST; night: 22:00–04:59 CEST).

In mixed models, “bird id” was used as random factor to avoid pseudoreplication. For each response variable, we conducted a random effect model with “colony” as second random factor (1) to make robust assumptions at the population level and (2) to correct for multiple testing (Gelman et al. 2012). In a second step, we used colony size, colony distance from the mainland, and both proportion variables as fixed effects to test their effects on all considered response variables with “colony” and “bird id” as random factors.

We had only one measurement per bird for UD, ID, and RD areas including the relevant intracolonial overlaps, and these responses were therefore analyzed without bird id as random factor.

Study year had no influence on any response variable and was therefore excluded from further analyses. Sex (analyzed following Suh et al. (2011)) was not considered in this study, to maximize sample size (available samples *n*_birds_ = 50, no data from HE) and because the sex ratio was similar in all colonies (Generalized Linear Model, all *P* > 0.15). Body mass had no effect on any foraging parameter (LM, *F*_5,49_ = 2.3, *P* = 0.1, *n*_birds_ = 58).

Helgoland might be seen as an outlier concerning its location about 45 km far off the nearest mainland coast (Fig. 2; Table S1), but not regarding the colony size. Therefore, we reran all models without HE. Although the exclusion of HE leads to changes in results of some parameters, we decided to leave HE birds in the analyses for the following reasons: (1) Excluding HE would mean losing 19 birds and thus produce a significant loss of data, which per se could influence the model output by weakening its explanatory power. (2) Without HE, there are only five random factor levels remaining for the random effect models. This can lead to a worse fit of the models, as, e.g., Gelman and Hill (2006) recommend at least five levels for random factors for adequate model estimation. All results without HE are reported in the supplementary material (Tables S6 and S7) to give a review of the resulting changes.

To provide a better comparison of effect sizes, the distance of each colony from the mainland and colony size were z‐transformed. Model prediction and credible interval (CrI) were estimated by simulating the posterior distributions (R package arm 1.7‐03) with 5000 simulations (Korner‐Nievergelt et al. 2015). To assume normality, proportions were arcsine‐transformed; maximum distance to nest, trip duration, and total distance flown were log‐transformed; and areas of 95% and 50% UD and 30% ID and RD were square‐root‐transformed. A Gaussian error distribution was used for all models. Visual inspection of residual plots found no obvious deviations of residual variances from homoscedasticity. Residuals of all models were independent and identically distributed (Korner‐Nievergelt et al. 2015).

We compared isotopic ratios among colonies using Stable Isotope Bayesian Ellipses in R (SIBER; R package siar 4.2), according to Jackson et al. (2011). We calculated the standard ellipse areas (SEAs) with correction for small sample sizes (SEAc, containing 40% of the data), the Bayesian estimate SEAb (number of posterior draws to make: 10,000), and the corresponding convex hull areas (TAs) according to Layman et al. (2007) following Jackson et al. (2011). LMs were performed to test for dependence of stable isotope values on colony size, colony distance from the mainland, and body mass.

## Results

### Flight and foraging patterns

Foraging trips of LBBGs breeding at the colonies close to the coast targeted both land and sea (Fig. 2). All foraging trips were spatially segregated among the studied breeding colonies (Fig. 2).

**Figure 3:**
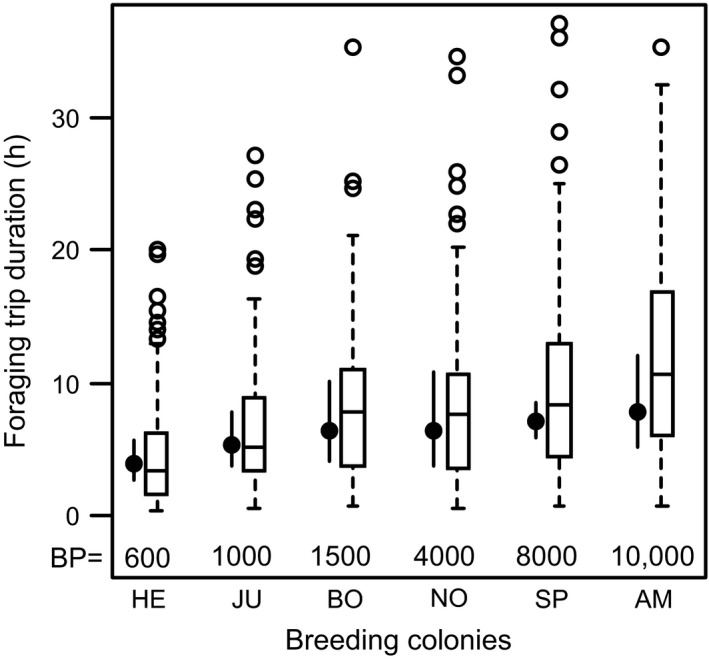
Intercolony comparison of foraging trip duration of all tracked *Larus fuscus* (*n* = 79) among different breeding colonies in the southern North Sea (BP = breeding pairs). White boxes contain 50% of the data (interquartile range). Whiskers represent the range of all observations that are <1.5 times the interquartile range away from the edge of the box5 the interquartile range above the upper whisker. The median is represented by the horizontal black line. Black dots represent the estimated group means including the symmetric 95% credible intervals (CrI) derived from LMMs.

**Figure 4:**
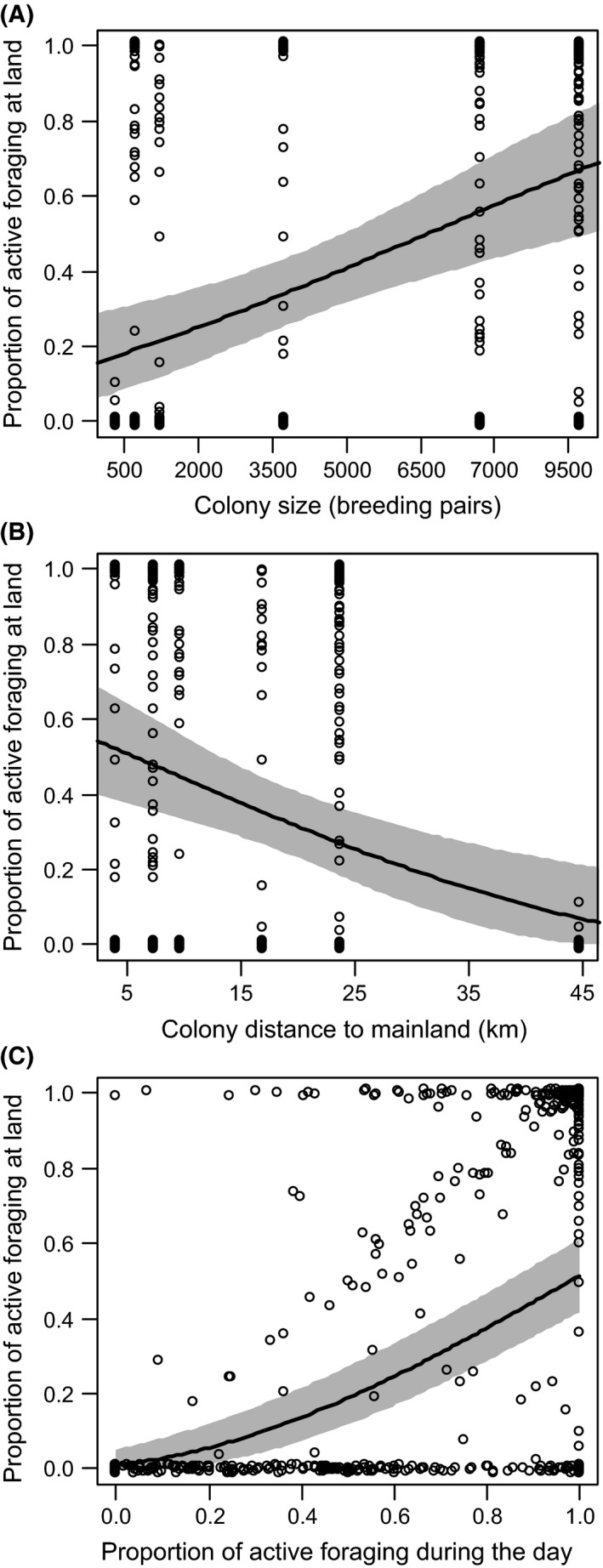
Proportion of active foraging at land/sea in relation to (A) colony size (estimated mean (95% CrI), 0.21 (0.07–0.36)); (B) distance of colony from the mainland (−0.18 (−0.33 to −0.03)); and (C) proportion of active foraging during the day/night (0.70 (0.58–0.80)) of all tracked *Larus fuscus* (*n* = 79). Residual SD was 0.50 (0.48–0.53), between‐colony SD was 0.12 (0.06–0.19), and between‐bird was SD 0.31 (0.27–0.35). Circles represent raw data, bold line represents predicted value for the population, and the symmetric 95% CrI is given in gray. When excluding birds from Helgoland, the effect of distance of colony from the mainland on terrestrial foraging disappears (Table S6).

Foraging patterns showed substantial differences among the colonies (Table 2). Birds from larger colonies made longer trips (Fig. 3) and travelled further than those from smaller colonies, independent of the distance of these colonies from the mainland (Table 3). Thus, maximum home range was greatest at the largest colonies. Likewise, active foraging was performed further away from the colonies with increasing colony size. Distance from the mainland was again only slightly relevant in these birds (Table 3). When comparing terrestrial and marine foraging, the gulls used more distant foraging patches at land than at sea (Table 3). The proportion of foraging at land increased with increasing colony size (Fig. 4A), and decreased with increasing distance from the mainland (Fig. 4B). That means the larger and the closer the colonies are to the mainland coast, the higher the amount of terrestrial foraging. LBBGs mostly foraged during the day, especially during terrestrial foraging (Fig. 4C). Foraging during the day was also carried out closer to the breeding colonies compared to nocturnal foraging, regardless of the colony size and the distance from the mainland (Table 3). Trips were generally longer at night than during the day and longer over land than over sea.

**Table 2:** Considerable colony‐specific differences in foraging trip parameters for *Larus fuscus* (*n*_trips_ = 838, *n*_birds_ = 79; areas of 30% ID and RD: *n*_trips_ = 811, *n*_birds_ = 78, stable isotope values: *n*_birds_ = 49) derived from linear mixed models. Colony‐specific estimated means and credible intervals including the variance parameters are given in the supporting information (Table S2)

	AM	BO	HE	JU	NO	SP
AM	–	Max. distance to nest Prop. of foraging at land *δ* ^13^C *δ* ^15^N	Trip duration Total distance travelled Max. distance to nest Prop. of foraging at land 95% UD area 50% UD area 30% ID area 30% RD area	Trip duration Total distance travelled Max. distance to nest 30% ID area *δ* ^15^N	30% ID area *δ* ^13^C *δ* ^15^N	*δ* ^13^C *δ* ^15^N
BO		–	Trip duration 95% UD area 50% UD area 30% ID area 30% RD area	*δ* ^13^C *δ* ^15^N		Max. distance to nest Prop. of foraging at land
HE			–	Prop. of foraging at land Prop. of foraging during the day	Trip duration Prop. of foraging at land	Trip duration Total distance travelled Max. distance to nest Prop. of foraging at land 95% UD area 50% UD area 30% ID area 30% RD area
JU				–	Prop. of foraging during the day	Total distance travelled Max. distance to nest 30% RD area
NO					–	
SP						–

**Table 3:** Foraging trip parameters for *Larus fuscus* (*n*_trips_ = 838, *n*_birds_ = 79; areas of 30% ID and RD, intracolonial UDOI of 95% UD: *n*_trips_ = 824, *n*_birds_ = 78). Relevant effects of predictors on the tested response variables including the effect sizes and symmetric credible intervals derived from linear mixed models. Effects of all other predictors and all corresponding variance parameters are given in the supporting information Tables S4, S5. Results excluding the breeding colony Helgoland are reported in the supporting information Tables S6, S7

Response variable	Relevant predictor	Effect size (95% CrI)
Trip duration (h)	Colony size	0.15 (0.05 to 0.26)
	Proportion of foraging at land	0.24 (0.42 to 0.59)
	Proportion of foraging during the day	−0.46 (−0.24 to −0.02)
Total distance travelled (km)	Colony size	0.26 (0.14 to 0.37)
	Proportion of foraging at land	0.17 (0.002 to 0.35)
Maximum distance to nest (km)^a^	Colony size	0.23 (0.11 to 0.35)
	Distance from the mainland	0.12 (0.01 to 0.22)
	Proportion of foraging at land	0.85 (0.71 to 0.99)
	Proportion of foraging during the day	−0.19 (−0.36 to −0.02)
Proportion of foraging during the day^a^	Proportion of foraging at land	0.45 (0.37 to 0.53)
95% UD area (km²)	Colony size	2.53 (1.01 to 4.04)
	Distance from the mainland	−1.73 (−3.31 to −0.20)
	Proportion of foraging at land	−2.03 (−2.99 to −1.10)
50% UD area (km²)	Colony size	1.01 (0.31 to 1.69)
	Distance from the mainland	−0.85 (−1.55 to −0.12)
	Proportion of foraging at land	−3.20 (−6.10 to −0.30)
30% ID area (km²)	Colony size	0.74 (0.15 to 1.34)
30% RD area (km²)	Colony size	1.04 (0.45 to 1.61)
	Distance from the mainland	−0.65 (−1.24 to −0.06)
	Proportion of foraging at land	−3.36 (−5.76 to −0.98)
Intracolonial UDOI of 95% UD	Proportion of foraging at land	0.03 (0.01 to 0.06)
*δ* ^13^C (‰)^b^	Proportion of foraging at land	−7.47 (−9.48 to −5.42)
*δ* ^15^N (‰)^b^	Proportion of foraging at land	3.70 (0.49 to 6.80)
	Proportion of foraging during the day	−7.73 (−9.85 to −5.50)

a Parameters of active foraging: *n*_trips_ = 811, *n*_birds_ = 78.

b Stable isotope values: *n*_birds_ = 49.

### Utilization distribution

UD, ID, and RD areas increased with increasing colony size (Table 3), i.e., birds from the largest breeding colonies also had the largest home ranges. UD and RD areas decreased with the increasing colony distance from the mainland, and the proportion of time spent flying at land. Areas used for commuting were thus smaller when LBBGs flew over land. In contrast, areas used for actual foraging (ID areas) were similar in size when flying over land or sea independent of the colony location (Table 3). Area sizes did not differ between day and night (Table 3).

**Figure 5:**
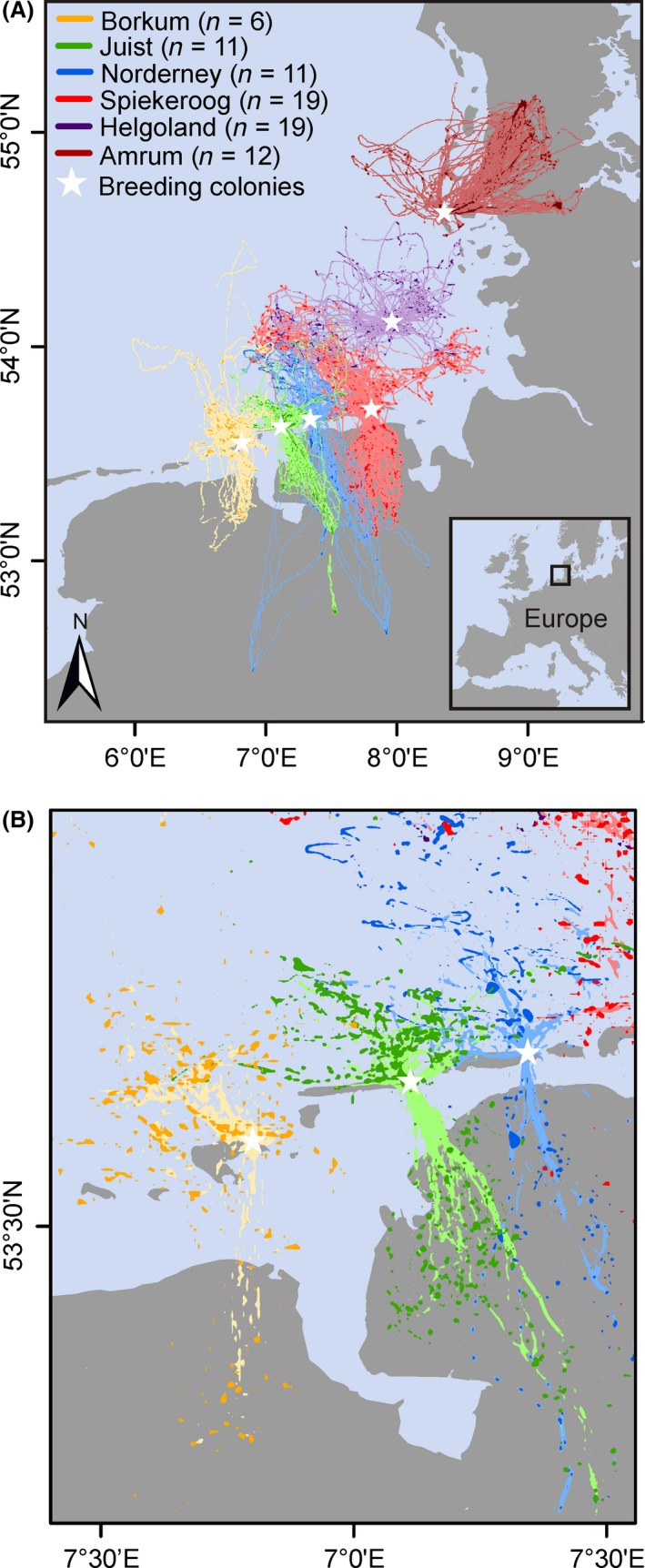
(A) Areas of 95% (light‐shaded colors) and 50% (dark‐shaded colors) utilization distribution; and (B) enlarged section of areas of 30% recursion distribution (light‐shaded colors) and intensity distribution (dark‐shaded colors) of all tracked *Larus fuscus* (*n* = 78) in the southern North Sea. White stars represent the location of the breeding colonies. Different colors indicate birds from different breeding colonies.

As the colony‐specific flight patterns were spatially segregated, there was little overlap among the colony‐specific UD, RD, and ID areas (Fig. 5; Table S8): the average overlap between all colonies was ≤0.01. The maximum overlap occurred between the adjacent colonies on JU and NO (Fig. 5; Table S8), although the degree of overlap was not significantly related to the distance between the relevant colonies (one‐way ANOVA, *F*_1,8_ = 3.421, adj. R² = 0.212, *P *=* *0.102, *n* = 10). Intracolonial overlaps of all utilization distribution areas (i.e., overlaps between all individuals of one colony) were generally low (Table S5), and unaffected by colony size, colony location, habitat type, and time of day (supported by low effect sizes; Table S4).

### Dietary analyses

**Figure 6:**
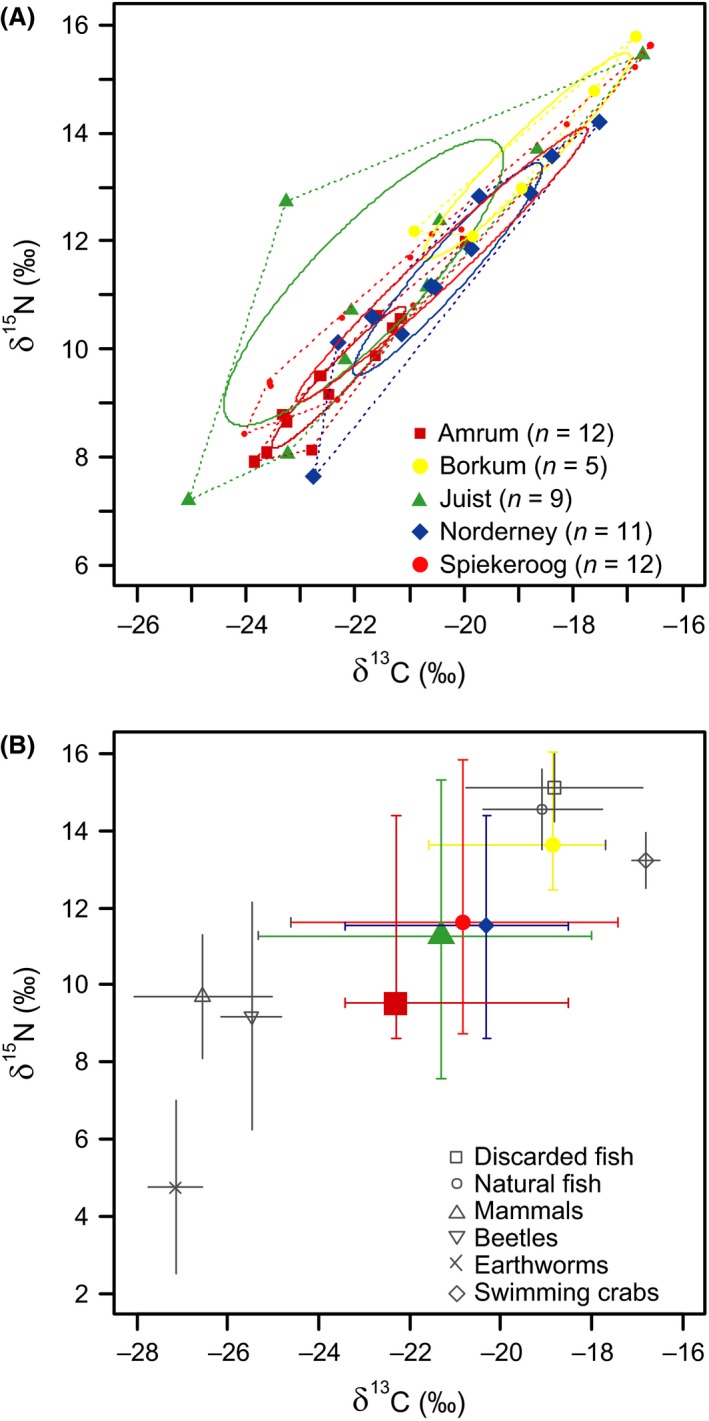
(A) Standard ellipse areas corrected for small sample sizes (SEAc, 40% credible interval) following Jackson et al. (2011) based on stable isotope values (*δ* ^13^C and *δ* ^15^N) in red blood cells and (B) colony‐specific isotopic values (mean ± SD) of *Larus fuscus* (*n* = 49) from different breeding colonies during the incubation periods from 2010 to 2013. Dotted lines indicate the layman metric of the convex hull area (TA). Different colors indicate different breeding colonies including all study years.

Pellet and stable isotope analyses demonstrated a varied dietary composition among the colonies ranging from earthworms and insects to fishes (mostly discard) and swimming crabs (*Liocarcinus* sp.), indicating both a broad range of marine and terrestrial prey items and the use of prey from low as well as high trophic levels (Fig. 6; Tables S9, S10). Dietary diversity (H') did not differ significantly among the colonies or among years (Kruskal Wallis Test, *χ*² = 8, df = 8, *P *=* *0.43). *δ* ^13^C and *δ* ^15^N ratios were unaffected by colony size and distance from the mainland (Table S4). Likewise, SIBER analyses revealed overlapping SEAcs for most colonies, except for birds from the two most distant ones (Fig. 6B; Table S10).

## Discussion

We aimed to identify and explain the diverse foraging behaviors of LBBGs from different colonies by tracking individuals from six breeding colonies of varying size and distance from the mainland. LBBGs showed colony‐specific foraging behaviors in terms of spatially segregated flight and foraging patterns, which were influenced by colony size, foraging habitat (marine or terrestrial), and partly by the colony's distance from the mainland.

### Spatial segregation and habitat use

LBBGs flew further and for longer and foraged further from their breeding colonies when colony size, and thus density‐dependent competition, was large. Likewise, they increased their UD areas. These results are in accordance with the findings of Ashmole (1963) reporting that larger colonies have larger foraging ranges. LBBGs increased their foraging range and used more distant foraging areas in response to intraspecific competition, not only from individuals within the same colony, but also from those at neighboring colonies. The low intercolonial overlap of ID further supports these findings and indicates the use of clearly segregated foraging sites, according to the findings of Cairns (1989) and Wakefield et al. (2013), despite the closeness of the colonies to each other.

The low the intracolony overlaps between the single individuals indicate that LBBGs do not only avoid intraspecific competition from neighboring colonies, but also from conspecifics within the same colony. This is in line with a recent study on Cory's shearwaters (*Calonectris borealis*) by Ceia et al. (2014), who reported at least partial segregation between foraging trips of individuals from two adjacent subcolonies. For LBBGs, the role of individual specialization in foraging sites needs further investigation.

### Foraging patterns

Spatial segregation of LBBGs, most likely caused by density‐dependent competition, was expressed not only by longer and further trips from the relevant breeding colony, but also by foraging in different habitats. Larger colony size was associated with a higher proportion of terrestrial foraging. As expected, individuals from coastal colonies tended to forage at land rather than at sea. In addition to avoiding intraspecific competition, the increase in terrestrial foraging might also reflect a continuously progressing food shortage at sea (Votier et al. 2004; Bertrand et al. 2012). This is further supported by Camphuysen et al. (2010) who found an increase in consumption of mammalian prey by herring gulls (*L. argentatus*) and LBBGs and predicted an increase in inland breeding for both species as a result of the apparent food shortage at sea. Following Bicknell et al. (2013), the amount of discards from fishing vessels, which represents an important food source for LBBGs (Camphuysen 1995; Garthe and Hüppop 1998), has also decreased in recent years, which could have an impact on the foraging behavior, as well as the spatial distribution of scavenging seabirds (Votier et al. 2013; Bodey et al. 2014a). The importance of discards for LBBGs might also be reflected by the pellet analyses of HE individuals, which demonstrated a switch from mostly discarded fish in 2009 to predominantly swimming crabs in 2011, probably associated with a 4‐week strike of the German, Dutch, and British shrimp fishermen during our study period in 2011.

Foraging at night is only possible in illuminated places, e.g., behind fishing vessels (Garthe and Hüppop 1996), because LBBGs hunt visually (Glutz von Blotzheim and Bauer 1982). Thus, terrestrial foraging was mostly performed during the day, while birds flew to sea during nocturnal foraging.

Possible advantages of terrestrial foraging might include the relatively uniform distribution of prey such as insects and earthworms in defined habitat types (Palm et al. 2013; Hackenberger and Hackenberger 2014). Together with their limited mobility, this suggests that terrestrial prey might reduce the foraging efforts required by gulls. The uniform distribution of terrestrial prey means that there is little competition for this food source, compared with marine prey, which is unpredictably and patchily distributed (Weimerskirch 2007), apart from the case of discarded fish (Cama et al. 2012). Levels of intraspecific and interspecific competition during scavenging behind fishing vessels are high (Furness 1992; Garthe and Hüppop 1998) and require a certain assertiveness (Camphuysen 1995; Tasker et al. 2000).

Terrestrial foraging also presents challenges: Although the nutrient contents of marine and terrestrial prey are similar (Golley 1961; Hislop et al. 1991; Finke 2002), the gulls need to consume more prey items to meet their energy requirements. Gulls generally performed longer trips and foraged further from their breeding colonies at land, which might be related to higher flight costs and energy requirements over land according to the predominant flight mode chosen (see Baudinette and Schmidt‐Nielsen (1974) and Ellis (1984) for lower flight costs during gliding than during flapping). Increased flight costs could be compensated for by visiting already‐known terrestrial foraging sites with good feeding conditions. The gulls could have used terrestrial habitats closer to the colony, assuming that food availability was similar to more distant areas, thus saving time and energy. They appeared to focus on predictable sites such as landfills and a meat factory about 135 km from the breeding colonies, as supported by the relatively straight and narrow routes flown to these inland areas. Using these remote foraging sites might also save time and energy, because of the high predictability of the anthropogenic food sources. This pattern could also explain the smaller UD, ID, and RD areas at land compared with marine sites. LBBGs probably prefer to forage regularly at well‐known sites that promise easily available prey with high nutritional value, such as the landfill sites. However, foraging at these sites might also be competitive, and further analysis of foraging site utilization on the individual level would help to clarify this issue. Detailed habitat mapping during the tracking period would help to clarify the reasons why LBBGs choose to fly long distances to terrestrial foraging sites. Whether or not the distribution of LBBGs during the breeding period is generally shifted toward terrestrial habitats over the last decade remains unclear.

### Dietary segregation

Dietary analyses support the above findings. Most colonies had a similar diversity in diets, and only differed in the composition of prey. The isotopic ratios were independent of colony size and distance from the mainland. This suggests that LBBGs use segregated foraging sites in different habitats but that these sites showed similar prey availability within the two main foraging habitats (Ceia et al. 2015). However, dietary segregation might occur at the individual, rather than the colony level, as shown, e.g., for other gull species (Masello et al. 2013).

In summary, LBBGs show colony‐specific, spatially segregated foraging patterns during incubation. Dietary segregation at the colony level is rare, suggesting that LBBGs forage in spatially segregated sites, often in opposing habitats, but focus on similar prey within these habitats. The foraging differences help to avoid intraspecific competition and are related to colony size and/or distances from the mainland. Foraging behavior is also affected by foraging habitat, given that trips were both temporally and spatially longer and UD areas were smaller at land than at sea. Inland foraging might thus also help to avoid density‐dependent competition. Given that birds are particularly constrained in terms of their foraging trip duration and distance during the chick rearing compared with the incubation phase, further studies are needed to analyze foraging strategies throughout the annual cycle.

## Data Accessibility

Tracking data are archived at Movebank (https://www.movebank.org).

## Ethics Statement

All institutional and national guidelines for the handling and the equipment of birds were followed. Birds were caught, ringed, and equipped under licenses issued by the National Park Administration of the Wadden Sea National Park of Lower Saxony, the Lower Saxony State Office for Consumer Protection and Food Safety (file number: 33.14‐42502‐04‐11/0666), the State Agency for Agriculture, Environment and Rural Areas Schleswig‐Holstein and the Ministry of Energy transition, Agriculture, Environment and Rural Areas Schleswig‐Holstein, Germany (file numbers: V 312‐72241.121‐37 (34‐4/11), V 312‐7224.121‐37 (80‐6/13)). All animals were handled in strict accordance with good animal practice to minimize handling time and stress.

## Conflict of Interest

None declared.

## Supporting information


**Data S1.** Instrumentation effect.
**Table S1.** Beeline distances (km) between the study colonies.
**Table S2.** Colony‐specific foraging‐trip parameters for *Larus fuscus* (*n*_trips_ = 838, *n*_birds_ = 79; areas of 30% ID and RD: *n*_trips_ = 811, *n*_birds_ = 78) derived from linear mixed models.
**Table S3.** Colony‐specific comparison of raw data means, standard deviations (SD) and estimated means (est. mean) including the 95% credible intervals (CrI) of intra‐colonial utilization distribution overlap indices (UDOIs), and isotopic ratios (red blood cells, RBC) of *Larus fuscus* (*n* = 79; isotopic ratios: *n* = 49) of *Larus fuscus* derived from linear mixed models (LMMs).
**Table S4.** Comparison of effect sizes (est. mean) and symmetric 95% credible intervals (CrI) including the relevant variance parameters of foraging trip parameters for *Larus fuscus* (*n*_trips_ = 838, *n*_birds_ = 79; areas of 30% ID and RD: *n*_trips_ = 824, *n*_birds_ = 78) derived from linear mixed models (LMMs).
**Table S5.** Intra‐colonial comparison of est. means and symmetric 95% CrI of utilization distribution overlap indices (UDOI) and isotopic ratios (red blood cells, RBC) of *Larus fuscus* (*n*_birds_ = 78; isotopic ratios: *n*_birds_ = 49) derived from linear mixed models (LMMs).
**Table S6.** Comparison of effect sizes (est. mean) and symmetric 95% credible intervals (CrI) including the relevant variance parameters of foraging trip parameters for *Larus fuscus* (*n*_trips_ = 698, *n*_birds_ = 60; areas of 30% ID and RD: *n*_trips_ = 684, *n*_birds_ = 59) derived from linear mixed models (LMMs).
**Table S7.** Intra‐colonial comparison of est.
**Table S8.** Inter‐colonial utilization distribution overlap index (UDOI) of 95% and 50% utilization distribution (UD), 30% intensity distribution (ID) and recursion distribution (RD) of equipped *Larus fuscus*.
**Table S9.** Dietary composition (%) and diversity (H′) of prey items in pellets of *Larus fuscus*.
**Data S2.** Prey items of *Larus fuscus* used for stable isotope analyses (SIA)
**Data S3.** Stable isotope analyses
**Table S10.** Colony‐specific results of Stable Isotope Bayesian Ellipses In R (SIBER) of red blood cells from captured *Larus fuscus* (*n* = 49).
