# Supplementary material for: Varying foraging patterns in response to competition? A multicolony approach in a generalist seabird
Source: Ecol Evol. 2016 Jan 20;6(4):974–86. doi: 10.1002/ece3.1884 (PMC4761771; doi:10.1002/ece3.1884)
Supplement: Supplementary file 1 — Data S1. Instrumentation effect. Table S1. Beeline distances (km) between the study colonies. Table S2. Colony‐specific foraging‐trip parameters for Larus fuscus (n trips = 838, n birds = 79; areas of 30% ID and RD: n trips = 811, n birds = 78) derived from linear mixed models. Table S3. Colony‐specific comparison of raw data means, standard deviations (SD) and estimated means (est. mean) including the 95% credible intervals (CrI) of intra‐colonial utilization distribution overlap indices (UDOIs), and isotopic ratios (red blood cells, RBC) of Larus fuscus (n = 79; isotopic ratios: n = 49) of Larus fuscus derived from linear mixed models (LMMs). Table S4. Comparison of effect sizes (est. mean) and symmetric 95% credible intervals (CrI) including the relevant variance parameters of foraging trip parameters for Larus fuscus (n trips = 838, n birds = 79; areas of 30% ID and RD: n trips = 824, n birds = 78) derived from linear mixed models (LMMs). Table S5. Intra‐colonial comparison of est. means and symmetric 95% CrI of utilization distribution overlap indices (UDOI) and isotopic ratios (red blood cells, RBC) of Larus fuscus (n birds = 78; isotopic ratios: n birds = 49) derived from linear mixed models (LMMs). Table S6. Comparison of effect sizes (est. mean) and symmetric 95% credible intervals (CrI) including the relevant variance parameters of foraging trip parameters for Larus fuscus (n trips = 698, n birds = 60; areas of 30% ID and RD: n trips = 684, n birds = 59) derived from linear mixed models (LMMs). Table S7. Intra‐colonial comparison of est. Table S8. Inter‐colonial utilization distribution overlap index (UDOI) of 95% and 50% utilization distribution (UD), 30% intensity distribution (ID) and recursion distribution (RD) of equipped Larus fuscus. Table S9. Dietary composition (%) and diversity (H′) of prey items in pellets of Larus fuscus. Data S2. Prey items of Larus fuscus used for stable isotope analyses (SIA) Data S3. Stable isotope analyses Table S10. Colon [file ECE3-6-0974-s001.pdf]

Supporting information (Ecology and Evolution)

Varying foraging patterns in response to competition? A multicolony approach in a generalist seabird

Anna-Marie Corman[*a], Bettina Mendel[a], Christian C. Voigt[b] and Stefan Garthe[a]

[a] Research and Technology Centre (FTZ), University of Kiel, Hafentörn 1, 25761 Büsum, Germany

[b] Leibniz Institute for Zoo and Wildlife Research (IZW), Alfred-Kowalke-Straße 16, 10315 Berlin, Germany

*Corresponding author: anna.corman@ftz-west.uni-kiel.de

Data S1: Instrumentation effect

We compared body masses at capture and recapture (available for only 58 birds). We also monitored all clutches periodically. It was not possible to conduct detailed behavioural observations of unequipped birds, and we therefore monitored the behaviours of all caught individuals after release and during the following days of the fieldwork period instead. The maximum time that an activated trap was left on the nest was 45 min, depending on the weather conditions.

Comparisons of the body masses of the equipped gulls at capture (mean ± SD: 818.3 ± 88.2 g, $n_{individuals}$ = 58) and recapture (784.2 ± 82.2 g) indicated an average body mass loss of 34.1 g (4.2%), which was not significant (Kruskal Wallis test, $\chi^2 = 2.2$, df = 1, $p = 0.1$, $n_{birds} = 58$). Thirty-eight individuals lost (range: 0.1–24.1%) and 17 gained body mass (range: 0.5–12.2%). Sixteen of the 103 equipped gulls (15.5%) lost their clutches as a result of predation, and two individuals refused to be captured while their clutch was covered with the trap. We confirmed chick hatching in the remaining individuals. The captured birds initially left the colony, but most

returned after 5–10 min to continue incubating. No abnormal behaviour was detected during observations of the equipped individuals throughout the capture period. Several equipped birds (n_{birds} = 34) were later identified during migration by reading their colour bands at their wintering grounds (multiple sightings at different locations in England, France, Spain, Portugal and Morocco) and/or at the breeding colonies in subsequent years. One individual from SP was equipped twice in two subsequent years with no evidence of abnormal behaviour. We therefore considered the effects of the GPS devices on the gulls to be low, though behavioural and energetic effects could not completely be excluded.

Table S1 Beeline distances (km) between the study colonies.

	AM-BO	AM-HE	AM-JU	AM-NO	AM-SP	BO-HE	BO-JU	BO-NO	BO-SP	HE-JU	HE-NO	HE-SP	JU-NO	JU-SP	NO-SP
**Beeline distance (km)**	162	63	140	129	112	100	20	40	69	80	65	47	18	47	18

Table S2 Colony-specific foraging-trip parameters for *Larus fuscus* (n_{trips} = 838, n_{birds} = 79; areas of 30% ID and RD: n_{trips} = 811, n_{birds} = 78) derived from linear mixed models. Considerable differences between colonies are indicated if the 95% credible intervals (CrI) of one colony do not contain the estimated mean (est. mean) of the other respective colony. Results for UDOIs are given in Table S3

**Colony**	**Statistical value**	**Trip duration (h)**	**Total distance travelled (km)**	**Maximum distance to nest (km)**	**Prop. of foraging at land**	**Prop. of foraging during the day**	**95% UD area (km²)**	**50% UD area (km²)**	**30% ID area (km²)**	**30% RD area (km²)**
**AM** (n = 13)	Mean	12.1	139.4	47.1	0.66	0.81	375.1	70.2	45.9	47.4
	SD	8.9	80.6	23.3	0.41	0.27	214.5	49.3	31.6	32.5
	Est. mean	7.9	96.0	33.2	0.69	0.88	304.5	55.8	34.3	37.6
	Lower CrI	5.2	61.8	20.8	0.21	0.78	214.5	38.8	23.7	25.2
	Upper CrI	12.0	151.2	53.6	0.99	0.95	410.5	75.6	47.1	51.6
**BO** (n = 6)	Mean	8.6	89.9	19.9	0.14	0.74	331.2	65.9	37.0	44.1
	SD	6.2	67.6	15.5	0.32	0.35	192.4	39.5	19.5	27.2
	Est. mean	6.5	65.5	16.3	0.09	0.86	272.8	52.3	29.5	35.7
	Lower CrI	4.2	42.1	10.2	0	0.76	167.7	32.3	17.8	21.9
	Upper CrI	10.0	104.3	26.5	0.51	0.94	396.3	77.3	44.7	52.4
**HE** (n = 19)	Mean	4.7	56.4	18.8	0.001	0.71	140.8	27.9	14.0	22.1
	SD	4.1	53.9	16.2	0.01	0.38	82.5	19.5	8.9	15.8
	Est. mean	3.9	44.9	15.4	0.002	0.83	151.4	29.6	15.0	23.0
	Lower CrI	2.7	30.9	10.0	0	0.74	99.1	19.4	9.5	15.4
	Upper CrI	5.7	66.6	23.7	0.24	0.91	212.6	41.9	21.7	32.4
**JU** (n = 11)	Mean	6.6	60.4	19.7	0.51	0.85	221.5	44.2	24.9	25.2
	SD	4.7	43.0	15.5	0.49	0.28	132.3	28.9	18.6	15.8
	Est. mean	5.4	51.7	15.1	0.49	0.91	214.7	41.9	22.5	26.3

	Lower CrI	3.7	35.8	9.8	0.11	0.84	139.3	26.9	14.0	16.5
	Upper CrI	7.8	74.9	23.2	0.88	0.96	303.8	60.0	33.0	38.6
**NO** (n = 11)	Mean	8.5	99.3	33.6	0.41	0.67	283.6	58.1	31.1	40.0
	SD	6.6	84.3	32.0	0.48	0.38	291.1	58.7	32.1	43.3
	Est. mean	6.5	68.7	21.6	0.41	0.81	219.2	44.0	23.1	30.7
	Lower CrI	3.9	39.5	11.6	0.002	0.68	141.1	29.0	14.3	20.2
	Upper CrI	10.9	120.8	40.2	0.95	0.91	312.4	62.7	33.3	43.5
**SP** (n = 19)	Mean	9.6	104.0	32.2	0.57	0.80	334.4	62.2	28.6	49.2
	SD	6.7	70.9	18.9	0.47	0.32	175.5	27.9	12.8	27.6
	Est. mean	7.1	80.6	26.9	0.59	0.89	293.3	55.2	26.2	41.4
	Lower CrI	6.0	66.4	21.7	0.44	0.84	219.5	41.1	18.8	31.0
	Upper CrI	8.5	97.8	33.3	0.74	0.93	374.6	71.5	34.9	53.4
**Variance paremeters**	Residual SD	0.77	0.75	0.74	0.55	0.47	4.45	2.59	1.99	2.21
	Lower CrI	0.74	0.71	0.71	0.52	0.44	4.25	2.24	1.72	1.90
	Upper CrI	0.81	0.79	0.78	0.58	0.49	4.67	3.05	2.33	2.60
	Between-colony SD	0.30	0.32	0.37	0.41	0.07	1.72	0.95	0.80	0.78
	Lower CrI	0.17	0.19	0.23	0.26	0.03	1.24	0.52	0.45	0.42
	Upper CrI	0.43	0.46	0.53	0.56	0.12	2.61	1.42	1.20	1.18
	Between-bird SD	0.33	0.39	0.44	0.33	0.13	X	X	X	X
	Lower CrI	0.29	0.34	0.40	0.29	0.11	X	X	X	X
	Upper CrI	0.38	0.44	0.50	0.36	0.15	X	X	X	X

Table S3 Colony-specific comparison of raw data means, standard deviations (SD) and estimated means (est. mean) including the 95% credible intervals (CrI) of intra-colonial utilization distribution overlap indices (UDOIs), and isotopic ratios (red blood cells, RBC) of *Larus fuscus* (n = 79; isotopic ratios: n = 49) of *Larus fuscus* derived from linear mixed models (LMMs). Areas of 30% ID and RD were analysed for 78 birds, because calculation failed for one individual from Amrum (AM). Considerable differences between colonies are indicated, if colony-specific CrI do not overlap with each other.

Colony	Statistical values	Intra-colonial UDOI of 95% UD	Intra-colonial UDOI of 50% UD	Intra-colonial UDOI of 30% ID	Intra-colonial UDOI of 30% RD	$\delta^{13}C$ (‰)	$\delta^{15}N$ (‰)
AM	Mean	0.08	0.008	0.0005	0.006	-22.36	12.22
	SD	0.11	0.010	0.0006	0.006	1.17	1.26
	Est. mean	0.05	0.006	0.0003	0.004	-21.97	12.60
	Lower CrI	0.04	0.004	0.0001	0.002	-23.02	11.49
	Upper CrI	0.07	0.008	0.0006	0.005	-20.91	13.64
BO	Mean	0.17	0.026	0.0015	0.014	-18.91	16.33
	SD	0.08	0.010	0.0012	0.008	1.64	1.65
	Est. mean	0.14	0.022	0.0006	0.011	-19.81	15.52
	Lower CrI	0.09	0.015	0.0002	0.007	-21.21	14.03
	Upper CrI	0.20	0.030	0.0012	0.016	-18.43	17.05
HE	Mean	0.04	0.006	0.0012	0.003	NA	NA
	SD	0.16	0.032	0.0127	0.014	NA	NA
	Est. mean	0.02	0.002	0.0001	0.001	NA	NA
	Lower CrI	0.01	0.001	0.0001	0.0004	NA	NA
	Upper CrI	0.03	0.003	0.0002	0.001	NA	NA

JU	Mean	0.12	0.014	0.0009	0.012	-21.36	13.98
	SD	0.12	0.015	0.0010	0.016	2.41	2.50
	Est. mean	0.09	0.009	0.0005	0.007	-21.21	14.02
	Lower CrI	0.07	0.006	0.0002	0.004	-22.38	12.81
	Upper CrI	0.11	0.012	0.0008	0.009	-20.10	15.21
NO	Mean	0.02	0.002	0.0003	0.002	-20.36	14.23
	SD	0.03	0.003	0.0005	0.003	1.64	1.87
	Est. mean	0.02	0.002	0.0002	0.001	-20.51	14.21
	Lower CrI	0.01	0.001	0.00003	0.0004	-21.57	13.08
	Upper CrI	0.03	0.003	0.0004	0.002	-19.41	15.35
SP	Mean	0.06	0.006	0.0011	0.005	-20.87	14.38
	SD	0.16	0.029	0.0116	0.014	2.67	2.53
	Est. mean	0.04	0.003	0.0002	0.003	-20.87	14.33
	Lower CrI	0.03	0.002	0.0001	0.002	-21.96	13.15
	Upper CrI	0.05	0.004	0.0003	0.013	-19.84	15.46
Variance parameters	Residual SD	0.15	0.058	0.027	0.047	2.02	2.06
	Lower CrI	0.14	0.054	0.026	0.044	1.68	1.71
	Upper CrI	0.16	0.060	0.029	0.050	2.50	2.56
	Between-colony SD	0.10	0.04	0.006	0.029	0.94	1.16
	Lower CrI	0.07	0.03	0.003	0.022	0.45	0.62
	Upper CrI	0.12	0.05	0.009	0.037	1.49	1.78

Table S4 Comparison of effect sizes (est. mean) and symmetric 95% credible intervals (CrI) including the relevant variance parameters of foraging trip parameters for *Larus fuscus* (n_{trips} = 838, n_{birds} = 79; areas of 30% ID and RD: n_{trips} = 824, n_{birds} = 78) derived from linear mixed models (LMMs). Predictors affected the single response variables if the CrI does not contain zero (bold).

**Predictor**	**Statistical value**	**Trip duration (h)**	**Total distance travelled (km)**	**Maximum distance to nest (km)**[a]	**Prop. of foraging during the day**[a]	**95% UD area (km²)**	**50% UD area (km²)**	**30% ID area (km²)**	**30% RD area (km²)**
Colony size	Est. mean	0.15	0.26	0.23	-0.05	2.53	1.01	0.74	1.04
	Lower CrI	**0.05**	**0.14**	**0.11**	-0.13	**1.01**	**0.31**	**0.15**	**0.45**
	Upper CrI	**0.26**	**0.37**	**0.35**	0.02	**4.04**	**1.69**	**1.34**	**1.61**
Distance from the mainland	Est. mean	-0.08	-0.03	0.12	0.04	-1.73	-0.85	-0.43	-0.65
	Lower CrI	-0.17	-0.14	**0.01**	-0.03	**-3.31**	**-1.55**	-1.06	**-1.24**
	Upper CrI	0.02	0.08	**0.22**	0.11	**-0.20**	**-0.12**	0.20	**-0.06**
Prop. of foraging at land	Est. mean	0.24	0.17	0.85	0.45	-2.03	-3.20	-1.59	-3.36
	Lower CrI	**0.42**	**0.002**	**0.71**	**0.37**	**-2.99**	**-6.10**	-3.95	**-5.76**
	Upper CrI	**0.59**	**0.35**	**0.99**	**0.53**	**-1.10**	**-0.30**	0.77	**-0.98**
Prop. of foraging during the day	Est. mean	-0.46	0.09	-0.19	X	1.39	1.27	0.50	0.35
	Lower CrI	**-0.24**	-0.13	**-0.36**	X	-6.88	-2.54	-2.55	-2.79
	Upper CrI	**-0.02**	0.29	**-0.02**	X	9.57	5.07	3.60	3.47
	Intercept	1.78	4.02	2.86	0.10	17.43	7.22	5.25	6.95
	Lower CrI	1.60	3.84	2.69	-0.07	11.82	4.63	3.12	4.77

	Upper CrI	1.97	4.22	3.03	0.28	23.00	9.82	7.35	9.13
Variance parameters	Residual SD	0.76	0.75	0.74	0.51	5.50	2.54	1.97	2.11
	Lower CrI	0.73	0.71	0.70	0.48	4.72	2.18	1.70	1.81
	Upper CrI	0.80	0.78	0.78	0.53	6.50	3.01	2.34	2.52
	Between-colony SD	0.04	5.5e-08	n.a.	0.12	1.42	0.38	0.76	5.2e-08
	Lower CrI	0.02	2.4e-08	n.a.	0.19	2.38	0.17	0.36	2.2e-08
	Upper CrI	0.07	1.1e-07	n.a.	0.06	0.63	0.67	1.25	9.0e-08
	Between-bird SD	0.35	0.41	0.45	0.31	X	X	X	X
	Lower CrI	0.30	0.36	0.39	0.27	X	X	X	X
	Upper CrI	0.40	0.47	0.51	0.35	X	X	X	X

[a]parameters of active foraging: n_{trips} = 811, n_{birds} = 78

Table S5 Intra-colonial comparison of est. means and symmetric 95% CrI of utilization distribution overlap indices (UDOI) and isotopic ratios (red blood cells, RBC) of *Larus fuscus* (n_{birds} = 78; isotopic ratios: n_{birds} = 49) derived from linear mixed models (LMMs). NA = not available, n.a. = not assessable (i.e., SDs are so small that the model cannot estimate it properly). Predictors affect the single response variables decisively, if CrI do not contain zero (bold).

**Predictor**	**Statistical values**	**Intra-colonial UDOI of 95% UD**	**Intra-colonial UDOI of 50% UD**	**Intra-colonial UDOI of 30% ID**	**Intra-colonial UDOI of 30% RD**	**$\delta^{13}C$ (‰)**	**$\delta^{15}N$ (‰)**
Colony size	Est. mean	-0.04	-0.02	-0.003	-0.010	-0.09	-0.22
	Lower CrI	-014	-0.04	-0.006	-0.030	-0.55	-0.70
	Upper CrI	0.06	0.01	0.001	0.010	0.37	0.25
Distance to mainland	Est. mean	-0.02	-0.004	-0.002	-0.008	-0.28	-0.34
	Lower CrI	-0.14	-0.04	-0.005	-0.031	-0.73	-0.82
	Upper CrI	0.10	0.03	0.002	0.015	0.17	0.13
Prop. of foraging during the day	Est. mean	0.06	0.04	0.018	0.033	2.80	3.70
	Lower CrI	-0.13	-0.03	-0.012	-0.026	-0.16	**0.49**
	Upper CrI	0.26	0.12	0.048	0.095	5.69	**6.80**
Prop. of foraging at land	Est. mean	0.08	0.03	0.009	0.032	-7.47	-7.73
	Lower CrI	-0.003	-0.004	-0.004	**0.005**	**-9.48**	**-9.85**
	Upper CrI	0.17	0.06	0.023	**0.059**	**-5.42**	**-5.50**
	Intercept	0.14	0.03	-0.004	0.016	-18.98	15.43
	Lower CrI	-0.04	-0.04	-0.025	-0.036	-20.80	13.41
	Upper CrI	0.31	0.09	0.019	0.066	-17.11	17.42

Variance parameters	Residual SD	0.15	0.057	0.027	0.047	1.37	1.48
	Lower CrI	0.14	0.054	0.026	0.045	1.12	1.21
	Upper CrI	0.16	0.061	0.029	0.050	1.71	1.85
	Between-colony SD	0.11	0.05	0.007	0.033	n.a.	n.a.
	Lower CrI	0.08	0.03	0.004	0.022	n.a.	n.a.
	Upper CrI	0.17	0.07	0.012	0.051	n.a.	n.a.

Table S6 Comparison of effect sizes (est. mean) and symmetric 95% credible intervals (CrI) including the relevant variance parameters of foraging trip parameters for *Larus fuscus* (n_{trips} = 698, n_{birds} = 60; areas of 30% ID and RD: n_{trips} = 684, n_{birds} = 59) derived from linear mixed models (LMMs). All LMMs were done excluding the breeding colony Helgoland. n.a. = not assessable (i.e., SDs are so small that the model cannot estimate it properly). Predictors affected the single response variables if the CrI does not contain zero (bold).

**Predictor**	**Statistical value**	**Trip duration (h)**	**Total distance travelled (km)**	**Maximum distance to nest (km)**[a]	**Prop. of foraging at land**[a]	**Prop. of foraging during the day**[a]	**95% UD area (km²)**	**50% UD area (km²)**	**30% ID area (km²)**	**30% RD area (km²)**
Colony size	Est. mean	0.10	0.21	0.25	0.17	-0.06	1.63	0.64	0.33	0.84
	Lower CrI	-0.02	**0.08**	**0.12**	**0.06**	-0.13	-0.17	-0.19	-0.35	**0.15**
	Upper CrI	0.22	**0.34**	**0.38**	**0.29**	0.01	3.51	1.47	1.00	**1.55**
Distance from the mainland	Est. mean	0.07	0.07	0.04	-0.10	0.07	1.50	0.49	1.18	0.10
	Lower CrI	-0.15	-0.16	-0.21	-0.31	-0.06	-2.16	-1.21	-0.19	-1.37
	Upper CrI	0.30	0.33	0.29	0.12	0.19	5.15	2.15	2.58	1.57
Prop. of foraging at land	Est. mean	0.59	0.36	0.89	X	0.45	-7.48	-3.28	-1.87	-3.31
	Lower CrI	**0.40**	**0.19**	**0.74**	X	**0.38**	**-14.88**	-6.69	-4.54	**-6.13**
	Upper CrI	**0.77**	**0.54**	**1.02**	X	**0.53**	**-0.15**	0.09	0.79	**-0.49**
Prop. of foraging during the day	Est. mean	-0.41	-0.06	-0.28	0.89	X	-0.25	0.70	-0.35	-0.28
	Lower CrI	**-0.66**	-0.30	**-0.47**	**0.75**	X	-11.40	-4.32	-4.45	-4.64
	Upper CrI	**-0.16**	0.18	**-0.10**	**1.02**	X	10.84	5.76	3.71	4.07
	Intercept	1.91	4.11	2.83	-0.001	1.03	21.23	8.62	7.13	7.92

	Lower CrI	1.68	3.87	2.61	-0.18	0.04	13.22	4.85	4.18	4.67
	Upper CrI	2.14	4.34	3.04	0.18	1.11	29.43	12.44	10.14	11.21
Variance parameters	Residual SD	0.74	0.71	0.74	0.54	0.40	6.01	2.71	2.14	2.29
	Lower CrI	0.71	0.68	0.71	0.51	0.38	5.03	2.27	1.79	1.91
	Upper CrI	0.79	0.75	0.78	0.57	0.43	7.34	3.33	2.62	2.82
	Between-colony SD	0.07	0.05	n.a.	0.14	0.08	n.a.	n.a.	n.a.	n.a.
	Lower CrI	0.03	0.02	n.a.	0.07	0.03	n.a.	n.a.	n.a.	n.a.
	Upper CrI	0.13	0.10	n.a.	0.23	0.14	n.a.	n.a.	n.a.	n.a.
	Between-bird SD	0.34	0.39	0.45	0.35	0.15	X	X	X	X
	Lower CrI	0.29	0.33	0.39	0.30	0.12	X	X	X	X
	Upper CrI	0.39	0.45	0.51	0.40	0.17	X	X	X	X

[a]parameters of active foraging: $n_{trips} = 681$, $n_{birds} = 59$

Table S7 Intra-colonial comparison of est. means and symmetric 95% CrI of utilization distribution overlap indices (UDOI) of *Larus fuscus* (n_{birds} = 60) derived from linear mixed models (LMMs) excluding the breeding colony Helgoland. The intra-colonial UDOIs of 30% ID and RD were analysed for 59 birds, because ID calculation failed for one individual from Amrum. Predictors affect the single response variables decisively, if CrI do not contain zero (bold).

**Predictor**	**Statistical values**	**Intra-colonial UDOI of 95% UD**	**Intra-colonial UDOI of 50% UD**	**Intra-colonial UDOI of 30% ID**	**Intra-colonial UDOI of 30% RD**
Colony size	Est. mean	-0.06	-0.02	-0.004	-0.016
	Lower CrI	**-0.09**	**-0.04**	**-0.007**	**-0.025**
	Upper CrI	**-0.02**	**-0.01**	**-0.001**	**-0.006**
Distance to mainland	Est. mean	0.06	0.03	0.004	0.015
	Lower CrI	**0.02**	**0.01**	**0.001**	**0.005**
	Upper CrI	**0.09**	**0.04**	**0.006**	**0.025**
Prop. of foraging during the day	Est. mean	0.10	0.03	0.017	0.043
	Lower CrI	-0.14	-0.05	-0.013	-0.032
	Upper CrI	0.34	0.12	0.048	0.118
Prop. of foraging at land	Est. mean	0.02	-0.002	-0.004	0.019
	Lower CrI	-0.07	-0.04	-0.018	-0.011
	Upper CrI	0.11	0.03	0.010	0.049
	Intercept	0.12	0.03	0.005	0.010

	Lower CrI	-0.05	-0.03	-0.019	-0.046
	Upper CrI	0.29	0.10	0.029	0.066
Variance parameters	Residual SD	0.15	0.054	0.025	0.050
	Lower CrI	0.14	0.051	0.023	0.046
	Upper CrI	0.16	0.059	0.026	0.053
	Between-colony SD	0.06	0.02	0.003	0.018
	Lower CrI	0.03	0.01	0.001	0.010
	Upper CrI	0.10	0.04	0.005	0.031

Table S8 Inter-colonial utilization distribution overlap index (UDOI) of 95% and 50% utilization distribution (UD), 30% intensity distribution (ID) and recursion distribution (RD) of equipped *Larus fuscus*. Areas of individuals from Amrum (AM) did not overlap with those of any other colony and are therefore not shown

**Colony**	**UDOI of 95 % UD**				**UDOI of 50 % UD**				**UDOI of 30 % ID**				**UDOI of 30 % RD**			
	**mean**	**SD**	**min**	**max**	**mean**	**SD**	**min**	**max**	**mean**	**SD**	**min**	**max**	**mean**	**SD**	**min**	**max**
BO-HE	0.0003	0.0007	0	0.0016	0	0.0001	0	0.0006	0	0	0	0.0003	0	0	0	0.0002
BO-JU	0.0002	0.0005	0	0.0020	0	0	0	0	0	0	0	0	0	0	0	0.0001
BO-NO	0.0003	0.0004	0	0.0014	0	0	0	0	0	0	0	0	0	0	0	0
BO-SP	0.0001	0.0001	0	0.0007	0	0	0	0.0001	0	0	0	0	0	0	0	0
HE-JU	0.0002	0.0005	0	0.0055	0	0	0	0.0001	0	0	0	0	0	0	0	0.0001
HE-NO	0.0003	0.0006	0	0.0040	0	0	0	0	0	0	0	0	0	0	0	0.0001
HE-SP	0.0001	0.0005	0	0.0075	0	0	0	0.0002	0	0	0	0.0001	0	0	0	0.0001
JU-NO	0.0097	0.0390	0	0.3670	0.0009	0.0035	0	0.0268	0.0001	0.0002	0	0.0017	0.0005	0.0033	0	0.0340
JU-SP	0.0017	0.0027	0	0.0170	0	0	0	0.0003	0	0	0	0.0003	0	0	0	0.0004
NO-SP	0.0027	0.0044	0	0.0314	0.0002	0.0004	0	0.0040	0.0001	0	0	0.0009	0.0001	0.0001	0	0.0017

Table S9 Dietary composition (%) and diversity (H’) of prey items in pellets of *Larus fuscus*. “Others” represents birds, eggs, grain and seeds. The grey scale represents a gradient from small (white) to high values (dark grey)

**Colony**	**Year**	**n**	**Bivalves**	**Bristleworms**	**Earthworms**	**Crabs**	**Insects**	**Fishes**	**Mammals**	**Waste**	**Others**	**H’**
AM	2011	160	11.4	3.8	4.2	59.9	6.8	5.8	3.3	0.6	4.1	1.21
AM	2012	122	16.3	1.6	12.3	30.9	18.1	4.6	2.3	1.6	12.4	1.74
BO	2012	93	29.2	0	15.4	30.7	12.0	6.1	5.0	0	1.6	1.70
HE	2009	144	0.1	0.9	0	24.8	0	65.5	0.7	1.4	5.6	0.91
HE	2011	87	1.1	0.3	0	79	0.4	16.8	2.1	0.2	0	0.61
JU	2013	129	7.2	2.0	23.4	11.6	27.2	5.4	8.1	7.2	7.8	1.77
NO	2013	121	0.5	1.2	33.5	5.0	34.2	3.4	5.3	3.5	13.4	1.55
SP	2010	45	5.3	3.8	10.4	10.2	14.2	28.0	11.3	8.9	7.8	2.88
SP	2012	131	0.8	0	35.8	8.9	31.6	5.2	4.2	1.3	12.2	1.49

H’ is calculated using a modification of the Shannon-Index (Shannon & Weaver 1949) $H' = -\sum p_i * \ln(p_i)$, where H′ is the diversity and p_i is the relative frequency of each dietary component in all pellets per colony. Borkum was a mixed colony with *Larus fuscus* and *L. argentatus*. The results of pellet analyses from this colony thus have to be interpreted with caution.

Data S2 Prey items of *Larus fuscus* used for stable isotope analyses (SIA)

Most important prey items derived from current and former pellet analyses (e.g. Kubetzki and Garthe 2003; Schwemmer and Garthe 2005) were classified into six groups of prey items:

- Naturally captured fish: *Clupea harengus*, *Sprattus sprattus*, *Callionymus lyra*
- Discarded fish: *Limanda limanda, Solea solea, Pleuronectes platessa, Gadus morhua, Merlangius merlangus, Eutrigla gurnardus, Trachurus trachurus, Belone belone*
- Swimming crabs: *Liocarcinus holsatus*
- Mammals: *Microtus arvalis, Arvicola terrestris, Talpa europaea*
- Insects: Staphilinidae sp., Carabidae sp.
- Earthworms: *Lumbricus sp*.

Marine prey items were caught during multi-year research vessel surveys of the Institute for Hydrobiology and Fishery Science (University of Hamburg/Germany) and the Institute of Sea Fisheries (Thünen Institute, Hamburg/Germany). Terrestrial prey items were collected from regurgitates during capture or recapture, or caught at areas similar to the terrestrial habitats the gulls used.

Data S3: Stable isotope analyses

Stable carbon and nitrogen isotope values of blood samples were measured simultaneously via continuous-flow isotope ratio mass spectrometry using a Flash Elemental Analyzer linked to a Delta V Advantage Isotope Ratio Mass Spectrometer (both Thermo Finnigan, Bremen, Germany). Two laboratory standards were analysed for every 10 unknown samples, allowing instrumental drift over a typical 14 h run to be corrected. Stable isotope ratios were expressed in δ notation as ‰ based on the international standards V-Pee-Dee Belemnite (carbon) and AIR (nitrogen), according to $\delta X = [(R_{sample}/R_{standard}) - 1] \times 1{,}000$ where X is ^{15}N or ^{13}C and R is the corresponding ratio $^{15}N/^{14}N$ or $^{13}C/^{12}C$. Based on internal standards (n = 165, tyrosin; Roth, Germany), the standard deviations were 0.16‰ and 0.29‰ for $\delta^{15}N$ and $\delta^{13}C$, respectively.

Table S10 Colony-specific results of Stable Isotope Bayesian Ellipses In R (SIBER) of red blood cells from captured *Larus fuscus* (n = 49). Area of the standard ellipse corrected for small ample sizes (SEAc, 40% credible interval), the Bayesian approximation of the standard ellipse area (SEAb) and the layman metric of convex hull area (TA) according to Jackson et al. (2011).

	AM (n = 12)	BO (n = 5)	JU (n = 9)	NO (n = 11)	SP (n = 12)
SEAb	1.26	2.50	9.66	2.91	3.65
SEAc	1.38	3.33	10.87	3.23	4.01
TA	2.61	2.38	19.62	6.23	6.29
